# *Camelina sativa* Oil Treatment Alleviates Castor Oil-Induced Diarrhea in ICR Mice by Regulating Intestinal Flora Composition

**DOI:** 10.1155/2022/5394514

**Published:** 2022-02-08

**Authors:** Jie Zhu, Liqin Yu, Yi Fan, Huanan Zhang, Feifei Li, Xiao Li, Yue Wei, Zhiyao Wang

**Affiliations:** ^1^Henan Napu Biotechnology Co., Ltd., Zhengzhou, Henan 450000, China; ^2^Henan Academy of Sciences, Zhengzhou, Henan 450000, China

## Abstract

Diarrhea, occurring due to intestinal flora disturbance, is potentially lethal, and its current treatments have adverse effects such as constipation and vomiting. *Camelina sativa* oil (CSO) is a cooking ingredient and natural remedy used in several countries; however, its pharmacological effects on intestinal health remain unknown. Here, we explored the CSO treatment effects on intestinal flora in male ICR mice with castor oil-induced diarrhea. The rate and degree of loose stools, the diarrhea index, serum inflammatory indices, fecal short-chain fatty acids (SCFAs), and the diversity and abundance of intestinal flora were measured. Castor oil-administered mice experienced diarrhea, reduced intestinal flora diversity and fecal SCFAs concentrations, altered intestinal flora composition, and increased serum proinflammatory indices. In contrast, CSO treatment relieved diarrhea, improved intestinal flora composition, and increased the relative abundance of *Lactobacillus* and *Lachnospiraceae*. Additionally, CSO significantly increased the concentrations of fecal propionic acid, valeric acid, isovaleric acid, and serum sIgA, while it reduced those of serum interleukin-17. These findings suggest that CSO could be a promising preventive agent against diarrhea.

## 1. Introduction

Diarrhea results from altered electrolyte absorption/secretion or nonabsorbable/osmotically active substances ingested from foods that accumulate in the intestinal lumen. It is a potentially lethal disease worldwide, particularly in infants, young children, and immune-compromised patients [1, 2], causing more than 0.5 million deaths of children annually [3, 4]. Long-term diarrhea in children can lead to malnutrition, growth retardation, and affect the immune system [5, 6]. In addition, diarrhea is also a common problem that affects up to 5% of adults [7]; this may lead to dehydration and have negative effects on the quality of life and work productivity [8].

A few clinical options to treat diarrhea include oral rehydration, antisecretory agents, probiotics, intestinal adsorbents, antibacterial and antiviral drugs, and the opioid receptor agonist loperamide [9]. Vomiting and constipation are the common adverse events of racecadotril and loperamide during the treatment of diarrhea; these cannot be effectively alleviated [10]. Antibiotics and antiviral treatments are intended to target specific pathogenic microorganisms; however, they pose considerable health challenges, which are extensive and unpredictable [11]. Therefore, to date, diarrhea treatment remains challenging, and there has been a continuous effort to identify avenues to stop diarrhea in a safe, simple, and inexpensive manner.

The intestinal flora is the “second genome” of the human body; it plays a vital role in the development of immune organs, human health, aging, and the occurrence of diseases. It ferments food to produce large quantities of short-chain fatty acids (SCFAs) that are pivotal signaling factors between the intestinal flora and the host [12–14]. Disturbances in the intestinal flora can lead to metabolic disorders, intestinal inflammatory diseases, metabolic syndrome, immune dysfunction, and other diseases [15, 16]. Intestinal flora and diarrhea-related diseases are closely related, including inflammatory bowel disease, malnourishment, and irritable bowel syndrome [17–19]. *ω*-3 polyunsaturated fatty acids (*ω*-3 PUFAs), such as eicosapentaenoic acid (EPA) and docosahexaenoic acid (DHA), are essential nutrients for human beings. These are synthesized only minimally in mammals; therefore, they must be obtained through dietary sources. *ω*-3 PUFAs exert potent anti-inflammatory effects via modulating membrane structure, attenuation of nuclear factor-kappa B (NF-*κ*B) activation, and stimulation of peroxisome proliferator-activated receptors, resulting in the reduced production of inflammation-related factors [20]. *ω*-3 PUFAs could modulate the diversity and composition of the intestinal flora. A clinical study on *ω*-3 PUFAs discovered that the abundance of *Lactobacillus* in intestinal flora increases at least two-fold following intervention with 4000 mg of EPA and DHA daily for 8 weeks; however, these large doses cause considerable treatment-emergent adverse events, including heartburn and acid regurgitation, and particularly in relation to dyspeptic symptoms [21]. Notably, several clinical studies have attempted to treat diarrhea-related diseases using fish oil, which is rich in EPA and DHA; however, the findings are discrepant, and the efficacy of the treatment remains to be fully elucidated [22].

*Camelina sativa* is an oilseed plant common in Europe and Asia and belongs to the *Cruciferae* family. *C. sativa* oil (CSO) contains >35% of *α*-linolenic acid (ALA), a dietary precursor of EPA and DHA. *C. sativa* has good agronomic properties and is suitable for growth in cold and arid areas, making CSO economical and a readily available replacement for fish oil. Additionally, CSO is a novel cooking ingredient in the United States, Canada, and many other countries [23]. Moreover, concerns regarding the nutraceutical effects of CSO are few and only focused on its hyperlipidemic effect [24]; however, the effect of CSO on intestinal health remains obscure.

Castor oil is often used in experiments to induce diarrhea because of its reproducibility and stability [25, 26]. Castor oil stimulates intestinal mucosal cells and reduces the active absorption of Na^+^ and K^+^, thereby inducing intestinal inflammation and diarrhea [27, 28]. The present study was designed to investigate the effect of preventive CSO administration on the intestinal flora of mice with castor oil-induced diarrhea. Our findings may facilitate the development of CSO administration as an alternative measure to treat and prevent diarrhea.

## 2. Materials and Methods

### 2.1. Animals

**Figure 1:**
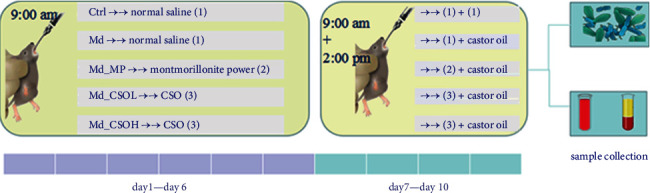
Experimental procedures.

Adult male (6-week-old) ICR mice (18–22 g) were purchased from Hunan Slake Jingda Experimental Animal Co., Ltd. (Changsha, China) and acclimatized to laboratory conditions for at least 1 week prior to the beginning of the experiments. Mice were kept in a room with a controlled temperature of 22–24°C, a stable humidity of 45–55%, and a 12-h light-dark cycle throughout the trial, and were allowed free access to water and food (standard mouse-breeding feed consisting of corn, soybean protein, flour, fish powder, brewer's yeast powder, vegetable oil, amino acids, calcium hydrogen phosphate, stone powder, salt, and compound vitamins; Beijing Huanyu Zhongke Biotechnology Co., Ltd., Beijing, China). The animal experiments were performed in accordance with the Regulations on the Administration of Experimental Animals issued by the Ministry of Science and Technology of China and approved by the animal welfare and ethic committee of Henan Napu Biotechnology Co., Ltd. (experiment approval number: HNP. No20210325002). An overview of the experimental procedures is shown in Figure 1.

### 2.2. Preventive Administration of CSO

CSO, which mainly contained 37.2% ALA, 16.6% linoleic acid, 15.8% eicosenoic acid, and 10.9% oleic acid, was produced in our laboratory from cold-pressed *Camelina* seed harvested in Henan province, China (CSO yield: 28.0%). Fifty mice (10 mice/group) were fed separately and randomly assigned to five groups: the control (Ctrl) and model (Md) groups were treated with 10 mL/kg body weight (BW) of normal saline; the CSO low-dose (Md_CSOL) and high-dose (Md_CSOH) groups were treated with 1.0 mL/kg BW and 2.0 mL/kg BW of CSO (equivalent to the recommended daily intake amounts for humans generally regarded as safe), respectively; and the positive control group (Md_MP) was treated with 3.0 g/kg BW of montmorillonite power (0.3 g/mL). All test substances were administered intragastrically at 9 : 00 am once daily for ten consecutive days.

### 2.3. Castor Oil-Mediated Induction of Diarrhea

Castor oil was administered to mice from day 7 of the experiment at 20 mL/kg BW for four consecutive days intragastrically at 2 : 00 pm to induce diarrhea. Normal saline, instead of castor oil, was administered to the Ctrl group. On day 10, after castor oil or normal saline treatment, the bedding in the cage was removed and replaced with filter paper of an appropriate size to observe feces, and the diarrhea status of mice was recorded for 5 h. The numbers of fecal and loose stool evacuations, as well as the rate of loose stool, degree of loose stool, and diarrhea index, were calculated according to a previously described method [29]. The rate of loose stool was defined as the percentage of the number of loose stools among the total fecal evacuations. The degree of loose stool indicated the level of loose stools and was graded according to the diameter of the stain formed by the loose stools on the filter paper. The degree of loose stool was rated as follows: (1) diameter of the stain <1 cm; (2) diameter of the stain ≥1 cm and <1.9 cm; (3) diameter of the stain ≥2 cm and ≤3 cm; and (4) diameter of the stain >3 cm. The longest and shortest diameters were measured for stains with irregular shapes, and the average was calculated. The diarrhea index was calculated as follows: the rate of loose stool × degree of loose stool.

### 2.4. Sample Collection and Processing

Fresh fecal samples were collected in sterile centrifuge tubes on day 10 and immediately stored at −80°C until further determination. Plasma was collected from the eyeball, separated from whole blood by centrifugation (1,200 × *g*) at 4°C for 10 min, and immediately stored at −80°C until subsequent determination.

### 2.5. High-Throughput Sequencing of Microbial 16S rDNA

Total microbial genomic DNA was extracted from the fecal samples (5 mice/Md_MP group and 6 mice/other groups) using the E. Z. N. ATM Mag-Bind soil DNA kit (Omega, Norcross, GA, USA). DNA quantity and concentration were measured using gel electrophoresis. The bacterial 16S rDNA V3–V4 variable region was amplified using forward primer 341F (5′-CCTACGGGNGGCWGCAG-3′) and reverse primer 805R (5′-GACTACHVGGGTATCTAATCC-3′). The DNA library was quantified using 2% agarose gel electrophoresis and a Qubit 3.0 fluorimeter (Thermo Fisher Scientific, Waltham, MA, USA) and sequenced on the Illumina MiSeqTM platform (Illumina, San Diego, CA, USA). The original data were filtered by removing primer connector sequences and low-quality bases; the effective data for each sample were obtained and used for further operational taxonomic unit (OTU) analysis.

### 2.6. SCFAs Analysis

Concentrations of five SCFAs (acetic, propionic, butyric, isobutyric, valeric, and isovaleric acids) in the feces were measured using gas chromatography. Fecal samples were accurately weighed and placed in 2-mL centrifuge tubes; methanol was added at a ratio of 1 : 5 (mg: *μ*L) and stirred with a homogenizer (IKA, Staufen im Breisgau, Germany) for 30 s until uniformly dispersed. Concentrated sulfuric acid was added to adjust the pH of the suspension to 2.0 to 3.0. After vortexing the mixture for 30 s and incubating for 10 min at ambient temperature, the samples were centrifuged (4,800 × *g*) at 4°C for 10 min. The supernatants were collected and injected into a gas chromatograph (aGC-2010 Plus; CD-WAX 30 m × 0.32 mm × 0.25 *μ*m; Shimadzu, Tokyo, Japan) with a flame ionization detector temperature. The oven temperature was set to 100°C, held for 2 min, and then raised to 200°C at 15°C/min (held for 10 min). The detector temperature was controlled at 260°C. The flow rates of make-up gas (N_2_), H_2_, and air were 3.0, 40, and 400 mL/min, respectively. A sample volume of 1.0 *μ*L was injected at 260°C using a split ratio of ∼5 : 1. The runtime for each sample was 15 min.

### 2.7. Inflammatory Index Determination

Proinflammatory indices, including those of interleukin (IL)-6, IL-1*β*, IL-17, interferon (IFN)-*γ*, and tumor necrosis factor-alpha (TNF-*α*), and the anti-inflammatory index of secretory immunoglobulin A (sIgA) were measured using commercial ELISA kits (Elabscience, Wuhan, China).

### 2.8. Correlation Analysis among Indices

We explored the relationships among indices, including SCFAs/inflammatory index, SCFAs/genus-level intestinal flora, SCFAs/SCFAs, and inflammatory index/inflammatory index, to investigate their respective interactions.

### 2.9. Statistical Analysis

Statistical analysis and result visualization were performed using SPSS (v.25.0) and R software (v3.6.0). Descriptive statistics were used for single-group data, and an independent sample *t*-test was used for two-group comparisons. Data were analyzed using either one-way analysis of variance and a post hoc LSD test for three-group comparisons when the data conformed to a normal distribution and the variance was homogeneous or Tamhane's T2 test when the data were nonnormally distributed. Differences were considered significant at *p* < 0.05. Spearman's correlation coefficients with 95% confidence intervals were performed to analyze the relationship between the parameters.

## 3. Results

### 3.1. Effects of CSO Treatment on Body Weights in Mice with Castor Oil-Induced Diarrhea

**Figure 2:**
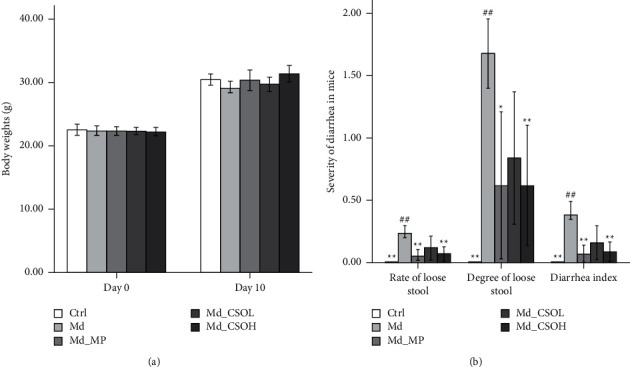
Influence of *Camelina sativa* oil (CSO) on the body weights of mice with diarrhea induced by using castor oil. (a) Body weights of mice in different groups; (b) severity of diarrhea in mice of different groups (vs model (Md) group, ^∗^*p* < 0.05, ^∗∗^*p* < 0.01; vs control (Ctrl) group, ^#^*p* < 0.05, ^##^*p* < 0.01).

We observed significant changes in mouse defecation, specifically loose stool, after treatment with castor oil, indicating the successful establishment of a murine diarrhea model. The feces excreted by mice in the Ctrl group were dry, granular, and soft throughout the experimental period. Mice in the Md group excreted soft, loose, or watery stool after the administration of castor oil for four days. Additionally, they showed gloomy spirits, with loose hair and little movement. The body weights of mice in the Md group on day 10 were slightly lower, and the rate of loose stool (0.23 vs. 0.00, *p* < 0.01), degree of loose stool (1.68 vs. 0.00, *p* < 0.01), and diarrhea index (0.38 vs. 0.00, *p* < 0.01) of mice in the Md group were markedly higher in comparison with those in the Ctrl group. The state of mood in mice from the Md_MP, Md_CSOL, and Md_CSOH groups improved, and the body weights of mice in these groups were slightly higher; the rate of loose stool (0.05 vs. 0.23, *p* < 0.01; 0.07 vs. 0.23, *p* < 0.01), degree of loose stool (0.62 vs. 1.68, *p* < 0.05; 0.62 vs. 1.68 *p* < 0.01), and diarrhea index (0.07 vs. 0.38, *p* < 0.01; 0.09 vs. 0.38, *p* < 0.01) of mice in the Md_MP and Md_CSOH groups were significantly lower than those in the Md group, indicating that CSO and MP treatments could effectively inhibit castor oil-induced diarrhea (Figures 2(a) and 2(b)).

### 3.2. Effects of CSO Treatment on Intestinal Flora Disturbance Induced by Castor Oil

**Figure 3:**
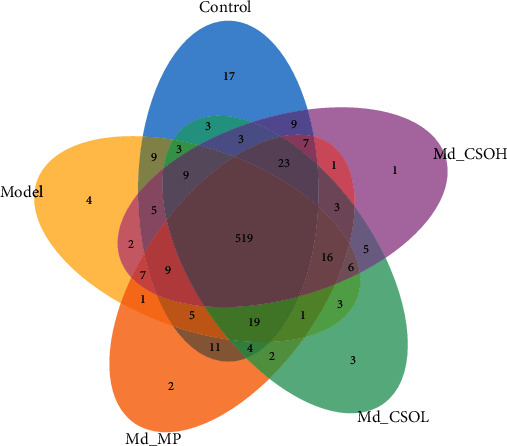
Venn chart of intestinal flora. Unique and common OTUs among different groups (control group (blue), model group (yellow), Md_MP group (orange), Md_CSOL group (green), and Md_CSOH group (pink)) as well as the common OTUs among them (gray).

The Venn diagram shows the number of common and unique OTUs and displays the diversity and overlap of OTU numbers in samples. There were 712 OTUs detected in samples, and OTU numbers in the Ctrl, Md, Md_MP, Md_CSOL, and Md_CSOH groups were 655, 619, 630, 622, and 625, respectively (Figure 3). There were 578 common OTUs in the Ctrl and Md groups, accounting for 88.24% of OTUs in the Ctrl group, implying that the administration of castor oil induced intestinal flora disturbance. There were 597, 583, and 584 common OTUs in the Ctrl and Md_MP, Md_CSOL, and Md_CSOH groups, accounting for 91.14%, 89.01%, and 89.16% of OTUs in the Ctrl group, respectively. This indicated that the preventive administration of MP and CSO alleviates the intestinal flora disorder induced by castor oil.

**Figure 4:**
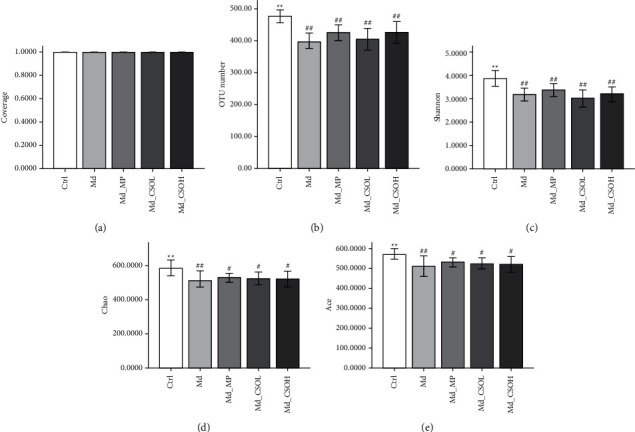
*α*-Diversity of mouse intestinal microbiota in different treatment groups. (a) Coverage; (b) OTU numbers; (c) Shannon; (d) Chao; and (e) Ace indices (vs model (Md) group, ^∗^*p* < 0.05, ^∗∗^*p* < 0.01; vs control (Ctrl) group, ^#^*p* < 0.05, ^##^*p* < 0.01).

*α*-Diversity is a critical parameter for investigating the diversity of intestinal flora, along with OTUs, Shannon, Chao, and Ace indices, the values of which are positively correlated with the diversity of intestinal flora in samples. The coverage index refers to the coverage of each sample library. The test result is approaching the actual sample if the coverage index is closer to 1. The average coverage index of each group was 1.0, indicating that the probability of undetected sequences in samples was low and that results were reliable (Figure 4(a)). The values of OTUs (395.33 vs. 476.67, *p* < 0.01), Shannon (3.18 vs. 3.88, *p* < 0.01), Chao (508.90 vs. 584.20, *p* < 0.01), and Ace (511.78 vs. 571.28, *p* < 0.01) indices in the Md group were significantly lower in comparison with those in the Ctrl group (Figures 4(b)–4(e)). This implied that castor oil administration markedly reduced the diversity of intestinal flora. The abovementioned indices were slightly higher with the preventive administration of CSO and MP, relative to those in the MP group; however, there was no significant difference.

### 3.3. Effects of CSO Treatment on the Phylum-Level Composition of Intestinal Flora

**Figure 5:**
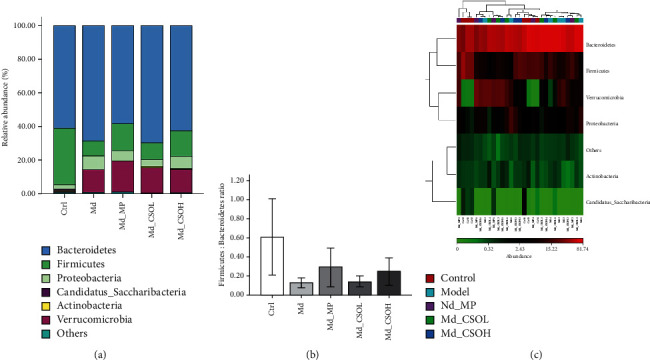
CSO altered the phylum-level composition of the intestinal flora. (a) Relative abundance of phyla in mouse intestinal flora; (b) Firmicutes-to-Bacteroidetes (F/B) ratios in different groups; (c) the phylum-level heatmap of samples.

Noticeable changes in the phylum-level composition of intestinal flora were observed after castor oil treatment (Figure 5). The Md group mice displayed a decreased relative abundance of the phylum Firmicutes (8.84% vs. 33.45%, *p* < 0.05) and Firmicutes: Bacteroidetes (F/B) ratio (0.13 vs. 0.61) and an increased relative abundance of the phylum Proteobacteria (8.19% vs. 2.76%) and Verrucomicrobia (13.51% vs. 0.48%) in comparison with those in the Ctrl group. Conversely, the relative abundance of Firmicutes and the F/B ratio in the Md_MP (16.11% vs. 8.84% and 0.29 vs. 0.13, respectively), Md_CSOL (9.91% vs. 8.84% and 0.14 vs. 0.13, respectively), and Md_CSOH (15.19% vs. 8.84% and 0.25 vs. 0.13, respectively) groups were slightly higher in comparison with those in the Md group; in contrast, the relative abundance of Proteobacteria was lower (6.25% vs. 8.19%, 4.18% vs. 8.19%, and 7.41 vs. 8.19%, respectively).

### 3.4. Effects of CSO Treatment on the Genus-Level Composition of Intestinal Flora

**Figure 6:**
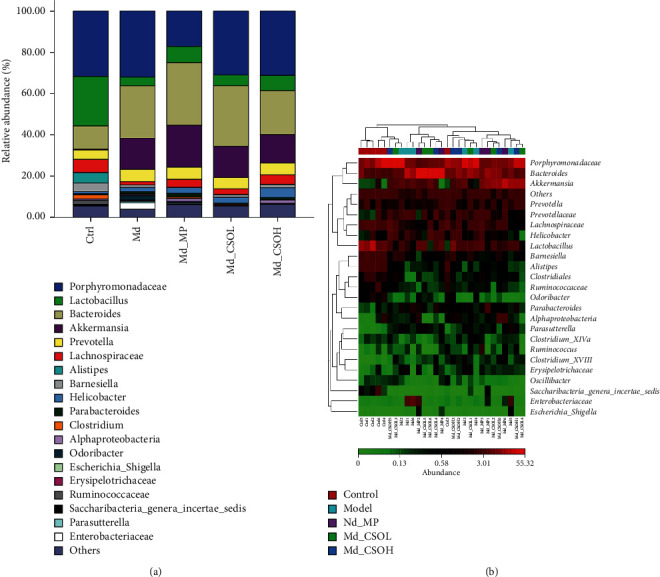
CSO altered the genus-level composition of the intestinal flora. (a) The relative abundance of the genus in mouse intestinal flora; (b) genus-level heatmap of samples.

The dominant genera in the intestinal flora were *Porphyromonadaceae*, *Lactobacillus*, *Bacteroides*, *Lachnospiraceae*, *Alistipes*, *Prevotella*, *Barnesiella*, and *Clostridiales* (*Clostridium*_XVIII, *Clostridium*_XlVa, and *Clostridiales*) in the Ctrl group, whereas those in the Md group included *Porphyromonadaceae*, *Bacteroides*, *Akkermansia*, *Prevotella*, *Lactobacillus*, and *Enterobacteriaceae*, accounting for >87% of the total genera (Figure 6). The relative abundance of *Lactobacillus* (4.21% vs. 24.01%, *p* < 0.01), *Lachnospiraceae* (2.00% vs. 7.44%, *p* < 0.01), and *Clostridiales* (0.78% vs. 2.13%, *p* < 0.01) was significantly decreased, and that of *Akkermansia* (13.45% vs. 0.47%) was increased in the Md group in comparison with those in the Ctrl group. The relative abundance of *Lactobacillus* and *Lachnospiraceae* in Md_MP (7.85% vs. 4.21% and 3.99% vs. 2.00%, respectively), Md_CSOL (5.21% vs. 4.21% and 2.95% vs. 2.00%, respectively), and Md_CSOH (7.38% vs. 4.21% and 4.48% vs. 2.00%, respectively) groups increased slightly but with no statistical difference in comparison with those in the Md group. Notably, *Alistipes*, *Ruminococcaceae*, *Oscillibacter*, and *Odoribacter* were detected in the Ctrl group but not in the Md group, whereas *Escherichia*, *Shigella* and *Erysipelotrichaceae* were detected only in the Md group.

### 3.5. Effects of CSO Treatment on SCFA Concentrations in Feces

**Figure 7:**
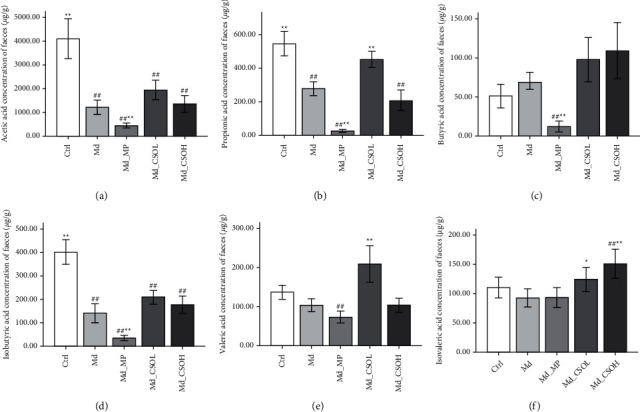
Fecal short-chain fatty acids (SCFAs) concentrations in different treatment groups. (a) Acetic acid; (b) propionic acid; (c) butyric acid; (d) isobutyric acid; (e) valeric acid; (f) isovaleric acid (vs model (Md) group, ^∗^*p* < 0.05, ^∗∗^*p* < 0.01; vs control (Ctrl) group, ^#^*p* < 0.05, ^##^*p* < 0.01).

Acetic acid was the most abundant SCFA in feces, followed by propionic, isobutyric, valeric, isovaleric, and butyric acids. The concentrations of acetic acid (1217.86 vs. 4105.70 mg/g, *p* < 0.01), propionic acid (278.84 vs. 547.86 mg/g, *p* < 0.01), and isobutyric acid (140.29 vs. 401.82 mg/g, *p* < 0.01) were significantly decreased in the fecal samples from castor oil-treated mice in the Md group in comparison with those in the Ctrl group (Figures 7(a), 7(b), and 7(d)). The concentrations of propionic acid (454.13 vs. 278.84 mg/g, *p* < 0.01), valeric acid (208.84 vs. 103.19 mg/g, *p* < 0.01), and isovaleric acid (124.08 vs. 92.68 mg/g, *p* < 0.05) were significantly increased (Figures 7(b), 7(e), and 7(f)), whereas those of other SCFAs, including acetic acid, butyric acid, and isobutyric acid, were slightly increased in the fecal samples of the Md_CSOL group in comparison with those in the Md group. The concentration of isovaleric acid (150.99 vs. 92.68 mg/g, *p* < 0.01) in fecal samples of Md_CSOH mice was markedly higher than that in the Md group (Figure 7(f)), indicating that CSO regulates the concentrations of intestinal SCFAs. Interestingly, MP treatment had no positive effect on SCFA concentrations; the amounts of acetic acid (446.96 vs. 1217.86 mg/g, *p* < 0.01), propionic acid (25.75 vs. 278.84 mg/g, *p* < 0.01), butyric acid (11.95 vs. 68.58 mg/g, *p* < 0.01), and isobutyric acid (35.27 vs. 140.29 mg/g, *p* < 0.01) were markedly lower than those in the Md group (Figures 7(a)–7(d)).

### 3.6. Effects of CSO Treatment on the Inflammatory Index

**Figure 8:**
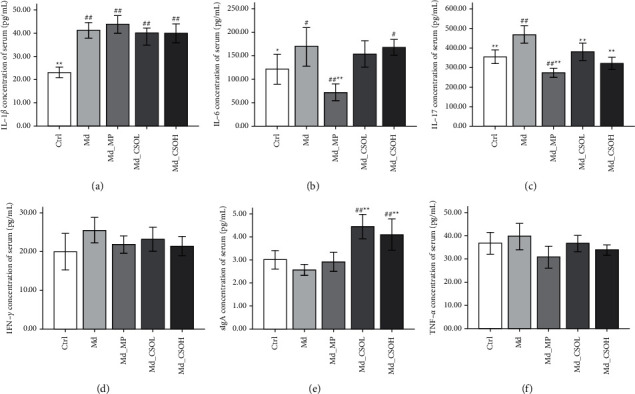
Inflammatory index in sera of mice in different treatment groups. (a) Interleukin 1*β* (IL-1*β*); (b) interleukin 6(IL-6); (c) interleukin 17 (IL-17); (d) interferon *γ* (IFN-*γ*); (e) secretory immunoglobulin A (sIgA); (f) tumor necrosis factor alpha (TNF-*α*) (vs model (Md) group, ^∗^*p* < 0.05, ^∗∗^*p* < 0.01; vs control (Ctrl) group, ^#^*p* < 0.05, ^##^*p* < 0.01).

Compared with those in the Ctrl group, IL-1*β* (41.21 vs. 23.09 pg/mL, *p* < 0.01), IL-6 (168.90 vs. 121.44 pg/mL, *p* < 0.05), and IL-17 concentrations (468.56 vs. 354.70 pg/mL, *p* < 0.01) in the sera from mice in the Md group were markedly elevated (Figures 8(a)–8(c)), whereas IFN-*γ*, TNF-*α*, and sIgA levels were not affected by the administration of castor oil (Figures 8(d)–8(f)). IL-17 levels in the sera of mice in the Md_CSOL (380.79 vs. 468.56 pg/mL, *p* < 0.01) and Md_CSOH groups (322.08 vs. 468.56 pg/mL, *p* < 0.01) were significantly decreased (Figure 8(c)), whereas sIgA levels were markedly increased in the Md_CSOL (4.44 vs. 2.56 pg/mL, *p* < 0.01) and Md_CSOH (4.10 vs. 2.56 pg/mL, *p* < 0.01) groups (Figure 8(e)), compared with those in the Md group. IL-6 (72.04 vs. 168.90 pg/mL, *p* < 0.01) and IL-17 (273.93 vs. 468.56 pg/mL, *p* < 0.01) levels in sera from mice in the Md_MP group decreased significantly, compared with those from mice in the Md group (Figures 8(b) and 8(c)).

### 3.7. Correlation Analysis

**Figure 9:**
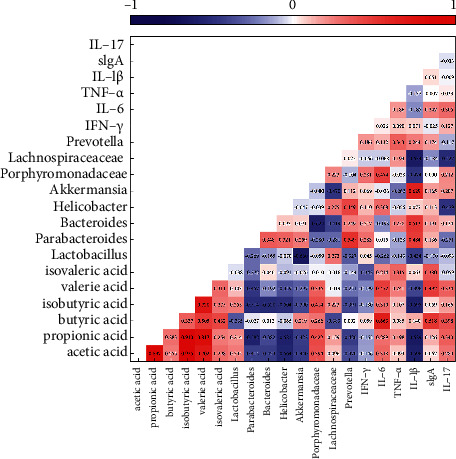
Heatmap of Spearman's correlation between short-chain fatty acids, genus level of intestinal flora, and the inflammation index (*n* = 29).

Heatmap visualization illustrated the correlation coefficients among SCFAs, relative abundance of genus-level intestinal flora, and inflammation indices (Figure 9). There were negative correlations between *Parabacteroides* (*p* < 0.05), *Bacteroides* (*p* < 0.05), *Helicobacter* (*p* < 0.01), *Akkermansia* (*p* < 0.05), and serum IL-1*β* (*p* < 0.01) and intestinal acetic acid levels. Additionally, we observed negative correlations between *Parabacteroides* (*p* < 0.01), *Helicobacter* (*p* < 0.01), *Akkermansia* (*p* < 0.05), *Prevotella* (*p* < 0.05), and serum IL-1*β* (*p* < 0.01) and intestinal propionic acid levels. Moreover, intestinal butyric acid levels were negatively correlated with *Lachnospiraceae* (*p* < 0.05); intestinal isobutyric acid levels were negatively correlated with the abundance of *Parabacteroides* (*p* < 0.01), *Bacteroides* (*p* < 0.01), *Helicobacter* (*p* < 0.01), *Prevotella* (*p* < 0.05), and serum IL-1*β* levels (*p* < 0.01), respectively. *Porphyromonadaceae* was significantly positively correlated with acetic acid (*p* < 0.05), propionic acid (*p* < 0.05), and isobutyric acid levels (*p* < 0.05). Furthermore, butyric acid and valeric acid were positively correlated with IL-6 (*p* < 0.01 and *p* < 0.05, respectively) and sIgA (*p* < 0.05 and *p* < 0.05, respectively). Notably, we observed a statistically significant correlation among SCFAs.

## 4. Discussion

In this study, we discussed the feasibility of the economic and convenient use of CSO to prevent diarrhea. We demonstrated that preventive CSO treatment markedly ameliorated castor oil-induced diarrhea in mice. The findings regarding CSO-specific mechanisms included the following: (i) improvement of the overall intestinal flora diversity; (ii) modulation of intestinal flora composition, especially elevating the relative abundance of *Lactobacillus* and *Lachnospiraceae*; (iii) enhancement of intestinal SCFA concentrations, including propionic acid and isovaleric acid; and (iv) decreased expression of proinflammatory factors along with increased sIgA expression.

The majority of previous *ω*-3 PUFA studies focused on their contributions to human health, including decreasing the risk of cardiovascular disease, breast and colorectal cancers, and promoting joint health. The tested substances were primarily seafood, especially fatty fish. The main components of them are EPA and DHA, which are often used as dietary supplements and ingredients of pharmaceutical preparations [30, 31]. Few studies have attempted to characterize ALA, a fat found in plant food, and its effect on intestinal diseases; small amounts of ALA can be metabolized to EPA and DHA after absorption. The main users and providers of *ω*-3 PUFAs are fatty fish; however, they are a finite and limited resource. Therefore, it is important to alleviate the global shortfall in these key nutrients through the agriculture industry [32]. Reviews on *ω*-3 PUFAs for the self-regulation of the inflammatory response and cardiovascular disease suggest that ALA differs from EPA and DHA in terms of the expression and activation of cyclooxygenase-2 and the outcomes of clinical studies [33, 34]. The fatty acid composition in plant oil is complex and includes *ω*-3 and *ω*-6 PUFAs, such as arachidonic acid, which is associated with promoting inflammation. Intake of *ω*-3 PUFAs through plant oil can lead to an increase in the amount of *ω*-6 PUFAs. Therefore, it is reasonable to speculate that investigating the impact of CSO on diarrhea not only expands the understanding of ALA and CSO but also promotes human health.

Our findings indicated that castor oil significantly decreased the diversity and composition of intestinal flora by decreasing the relative abundance of the phylum Firmicutes and increasing the relative abundance of Proteobacteria and Verrucomicrobia without affecting that of Bacteroidetes. Firmicutes and Bacteroidetes are the main intestinal phyla that regulate host inflammation and adaptive immunity. The F/B ratio is considered an indicator of health status, especially in the intestine [35]. An elevated F/B ratio indicates that the body is in a state of inflammation and immunological imbalance. Excessive growth of Firmicutes can produce metabolic endotoxins, such as lipopolysaccharides, that trigger systemic inflammation [25, 36]. However, in this study, we obtained a contradictory result; the F/B ratio decreased with diarrhea and intestinal inflammation. This was consistent with the results reported by Jacobs et al. [37]. The intestinal flora in both Crohn's disease and ulcerative colitis exhibits a general decrease in taxonomic diversity relative to that in the healthy intestinal flora. In addition, there is a phylum-level decrease in Firmicutes and an increase in Proteobacteria. The difference could be due to the different environmental influences, such as diets, and distinct species, including humans and mice. Firmicutes comprise several genera of outstanding relevance in healthcare, such as *Staphylococcus* and *Listeria*, which are pathogenic, and lactic acid bacteria, which are used as probiotics and in the preparation of food products [38]. Members of the genus *Lactobacillus* are associated with good intestinal health. *Lactobacillus* can inhibit pathogen colonization via the production of several acidic metabolites, mainly acetic acid and lactic acid [39]. The relative abundances of the dominant genera *Lactobacillus* and *Lachnospiraceae* were significantly decreased after the castor oil treatment, leading to a lower F/B ratio with diarrhea.

Furthermore, we found that the relative abundances of *Akkermansia*, *Bacteroides*, *Parabacteroides*, and *Helicobacte*r were negatively correlated with SCFAs and positively correlated with proinflammation indicators. The abundance of *Akkermansia* and *Bacteroides* positively correlates with proinflammation pathways, including the IFN signaling pathway and T cell and monocyte expression of NF-*κ*B [40], which was consistent with the results in this study. SCFAs participate in host immune regulation and enhance the intestinal barrier [41]. We observed positive correlations between SCFAs and sIgA, which represents the first line of mucosal immunity and is essential for the intestinal barrier. sIgA coating and steric hindrance can prevent microbial adhesins from contacting and interacting with the epithelium. sIgA can also specifically inhibit pathogens by directly recognizing receptor-binding domains, such as that of reovirus type 1 [42].

The connections between ALA and intestinal flora are not fully elucidated. Dietary *ω*-3 PUFAs (DHA and EPA) can improve the health of patients with inflammatory bowel disease. This is achieved by reverting the disordered intestinal flora to a healthy state by decreasing the F/B ratio, inhibiting the production of proinflammatory factors, such as TNF-*α* and IL-17, promoting the production of anti-inflammatory factors, such as IL-10, and increasing the content of SCFAs [43–46]. In this study, the preventive administration of CSO decreased the levels of the proinflammatory factor IL-17 and increased the levels of the anti-inflammatory factors, sIgA, and SCFAs. The discrepancy in the F/B ratio trend can be attributed to the different effects of ALA and DHA/EPA and the presence of several fatty acids (not only ALA) in CSO. Noriega et al. reported that supplementation with *ω*-3 PUFAs at a daily dose of 600 mg through a fish protein diet for 2 weeks leads to an increase in Firmicutes and a decrease in those of Bacteroidetes and Actinobacteria; this is consistent with the findings of this study [47].

## 5. Conclusions

CSO treatment could regulate intestinal flora and the expression of inflammatory factors. In addition, it improved the levels of SCFAs in the feces of mice, preventing or alleviating diarrhea. Therefore, CSO administration can be a promising strategy for preventing diarrhea. However, this study has several limitations. This study evaluated the CSO pretreatment of mice by treating them with CSO and then inducing diarrhea; therefore, the result is focused on the preventive effect of CSO. The efficacy of CSO for treating diarrhea remains uncertain. SCFAs exert anti-inflammatory effects, and the intestinal flora directly influences the availability of ALA; therefore, further studies are needed to determine whether the interaction between ALA and intestinal flora can enhance the anti-inflammatory effect of CSO.

## Data Availability

The data used to support the research are included within this manuscript.
